# Pediatrics Ewing's Sarcoma of the Sinonasal Tract: A Case Report and Literature Review

**DOI:** 10.1155/2019/8201674

**Published:** 2019-01-02

**Authors:** Ahmed Aldandan, Ali ‎ Almomen, Abdulrahman Alkhatib, Ghaleb Alazzeh

**Affiliations:** ^1^Imam Abdulrahman Bin Faisal University, Dammam, Saudi Arabia; ^2^Department of ENT, King Fahad Specialist Hospital, Ministry of Health, Dammam, Saudi Arabia

## Abstract

Ewing's sarcoma (ES) is a highly malignant, small, round cell tumor that originates from the primitive neuroectodermal cells. Primary ES commonly occurs in early childhood or adolescence. It may present with skeletal and extraskeletal forms. The extraskeletal form is rarely encountered in the head and neck region and is extremely rare in the sinonasal tract. This is a case report of sinonasal ES in a 13-year-old female patient who presented with a 7-month history of right nasal obstruction, anosmia, intermittent epistaxis, snoring, and hearing loss. Clinical examination revealed a right nasal mass pushing the septum to the left side and extending to the nasopharynx. Endoscopic biopsy and histopathological analysis showed a small blue cell tumor suggestive of ES. The patient was treated with surgery, radiotherapy, and chemotherapy. After a follow-up of 5 years, the patient remains recurrence-free with excellent functional status and quality of life.

## 1. Introduction

Ewing's sarcoma (ES) is a highly malignant and rare tumor that subdivided into skeletal and extraskeletal entities [1, 2]. The skeletal form is more common and typically involves the long bones of the extremities [1, 2]. The less common extraskeletal form involves soft tissues of the lower extremities, paravertebral tissues, chest wall, and retroperitoneum [1, 3]. It rarely affects the head and neck [1, 3]. Sinonasal ES is very rare and only few reported cases have been published in literature [1].

## 2. Case Report

On March 2010, a 13-year-old female patient referred to the ENT clinic complaining of right sided nasal obstruction, anosmia, intermittent epistaxis, snoring, and hearing loss for 7-month duration. There was no history of trauma, anorexia, or weight loss.

Clinical examination revealed a right sided nasal mass pushing the septum to the left side and extending to the nasopharynx. On throat examination, the soft palate was pushed down by the nasopharyngeal mass. Otoscopy showed dullness and retraction of tympanic membrane bilaterally. Cranial nerves examination was normal. No cervical lymph nodes were palpable. The results of hematological and biochemical investigations were within normal limits.

**Figure 1:**
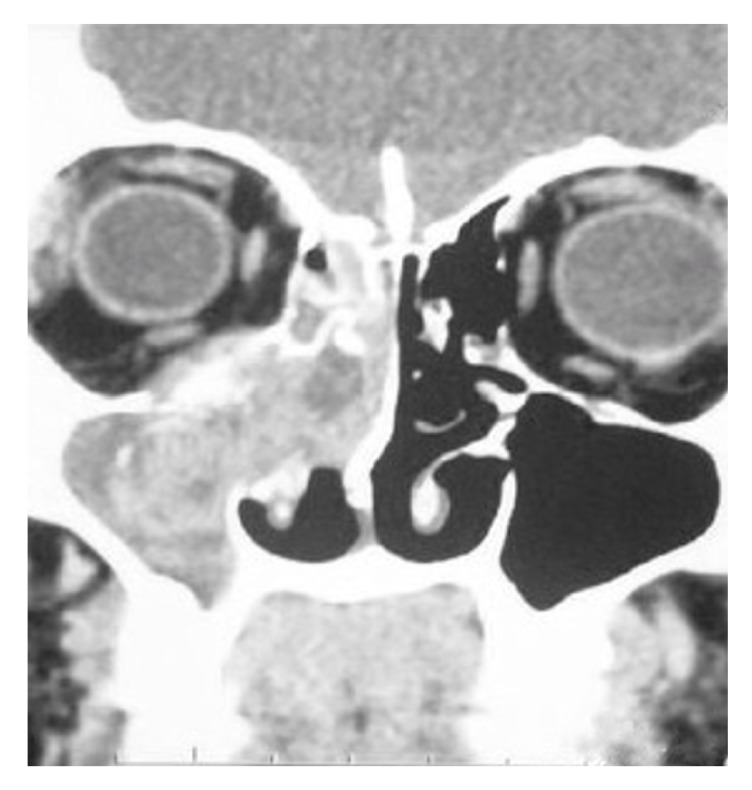
Computed tomography (CT) scan showing a mass causing an opacification of the right nasal cavity, ethmoid and maxillary sinuses.

**Figure 2:**
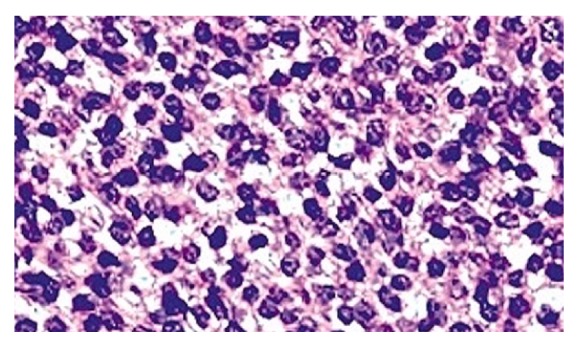
Microscopic analysis showing sheets of small round blue cells.

**Figure 3:**
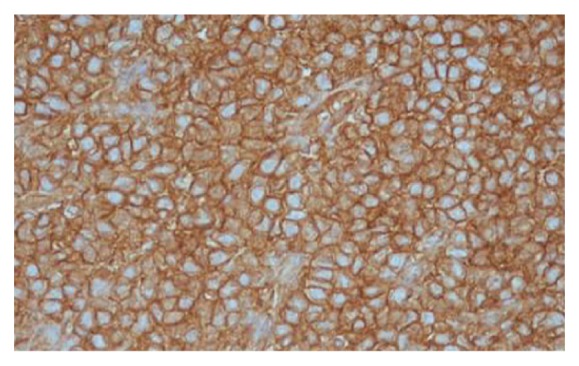
Immunohistochemistry of tumour sample showing CD99 positivity.

On radiological evaluation, CT scan revealed an opacification of the right nasal cavity, maxillary, ethmoidal, sphenoid, and frontal sinuses with bone remodeling of the septum to the left side (Figure 1). Subsequently, the patient underwent endoscopic excision of the tumor that was occupying the right nose, maxillary, ethmoid sinuses, and nasopharynx. The posterior ethmoid, sphenoid, and frontal sinuses were free of the disease. Histopathological analysis showed a small blue cell tumor (Figure 2). Immunohistochemistry showed the neoplastic cells are positive for CD99 marker (Figure 3). Molecular study using fluorescence in situ hybridization (FISH) had shown EWSR1 gene rearrangement in 100% of the analyzed nuclei that confirm the diagnosis of ES. The patient was treated with surgery, radiotherapy, and chemotherapy. After a follow-up of 5 years, the patient remains recurrence-free with excellent functional status and quality of life.

## 3. Discussion

ES is a highly malignant, small, round cell tumor that originates from the primitive neuroectodermal cells [4, 5]. It was first described by James Ewing in 1921 [1]. Primary ES commonly occurs in early childhood or adolescence and rarely occurs in adulthood [6]. Head and neck ES usually presents in patients younger than 30 years of age, with a peak incidence in those aged 10 to 15 years [6, 7]. ES has a slight male gender predominance with a male to female ratio of 1.5:1 [6, 8, 9].

ES is a rare disease that accounts for only 4% to 6% of all primary bone tumors [2, 7, 8]. Furthermore, ES involves the head and neck region in only 1% to 4% of cases, and primary sinonasal ES is even rarer [2, 8]. In the sinonasal tract, the differential diagnosis includes all tumors that are composed of small round cells, such as rhabdomyosarcoma, lymphoma, poorly differentiated carcinomas, melanoma, and olfactory neuroblastoma [1, 7, 10]. It is difficult to differentiate ES from these tumors based on clinical and radiological examination alone; hence it requires a histopathological examination, an immunohistochemistry, and a cytogenetic analysis to reach a definitive diagnosis [4]. The essential diagnostic test to differentiate ES from the many small round neoplasms is the CD99 marker, which can be detected in a specific immunohistochemical examination [2, 4]. Molecular analysis to detect chromosomal translocation can be used to confirm diagnosis of ES [2]. Most cases of ES are characterized by translocation of the Ewing's sarcoma gene (EWS), which is located on 22q12. EWS is fused with the friend leukemia virus integration site 1 gene (FLI-1), which is located on 11q24 [2, 5]. This fusion results in a t(11,22) translocation which is found in 85-90% of the cases [2, 5].

Clinical manifestations of sinonasal ES include enlarging mass, nasal obstruction, rhinorrhea, and epistaxis [5, 9]. Approximately 18% of the patients are presented with metastasis at time of diagnosis [6]. The most common sites of distant metastasis are the lungs and bones [5].

The prognosis depends on the site of the primary tumor, the presence of distant metastasis at presentation, and the age of the patient [1, 6]. Researchers have found that patients younger than 15 years of age and patients with axial and sinonasal tract diseases have a better prognosis [1, 6]. While the 5-year survival of patients with metastases is around 22%, it is 55% in those without metastases [1, 6, 9]. However, the effective treatment of ES has improved the survival rate up to 86% in patients without metastatic disease [7, 11].

Successful treatment of ES includes a multidisciplinary approach with surgery followed by adjuvant radiotherapy and chemotherapy [1]. The recommended chemotherapy regimen is alternating cycles of vincristine-doxorubicin-cyclophosphamide and ifosfamide-etoposide [12]. In fact, the Children's Oncology Group has demonstrated a 5-year event-free survival of 73% in patients with localized tumors treated with these drugs [12]. In our case, the patient responds well to the surgery, radiotherapy, and chemotherapy without any evidence of recurrence of the disease.

## 4. Conclusion

Ewing's sarcoma rarely affects the sinonasal tract. Diagnosis of the disease is challenging hence it requires histopathological examination, immunohistochemical studies, and cytogenetic studies. Treatment includes a multidisciplinary approach with surgery followed by chemotherapy and radiotherapy.

## References

[1] Yeshvanth S. K., Ninan K., Bhandary S. K., Lakshinarayana K. P. H., Shetty J. K., Makannavar J. H. (2012). Rare case of extraskeletal Ewings sarcoma of the sinonasal tract. *Journal of Cancer Research and Therapeutics*.

[2] Liang J. (2018). Sinonasal Ewing Sarcoma: A Case Report and Literature Review. *The Permanente Journal*.

[3] Negru M. E., Sponghini A. P., Rondonotti D., Platini F., Giavarra M., Forti L. (2015). Primary Ewings sarcoma of the sinonasal tract, eroding the ethmoid and sphenoid sinus with intracranial extension: A rare case report. *Molecular and Clinical Oncology*.

[4] Suzuki T., Yasumatsu R., Nakashima T., Arita S., Yamamoto H., Nakagawa T. (2017). Primary Ewing’s Sarcoma of the Sinonasal Tract: A Case Report. *Case Reports in Oncology*.

[5] Vaccani J. P., Forte V., de Jong A. L., Taylor G. (1999). Ewing's sarcoma of the head and neck in children. *International Journal of Pediatric Otorhinolaryngology*.

[6] Howarth K. L., Khodaei I., Karkanevatos A., Clarke R. W. (2004). A sinonasal primary Ewing's sarcoma. *International Journal of Pediatric Otorhinolaryngology*.

[7] Hafezi S., Seethala R. R., Stelow E. B. (2011). Ewing's family of tumors of the sinonasal tract and maxillary bone. *Head & Neck Pathology*.

[8] Balamuth N. J., Womer R. B. (2010). Ewing's sarcoma. *The Lancet Oncology*.

[9] Souheil J., Skander K., Sawssen D. (2016). Ewing sarcomas of the sino-nasal tract and maxillary bone. *Egyptian Journal of Ear, Nose, Throat and Allied Sciences*.

[10] Wenig B. M. (2009). Undifferentiated malignant neoplasms of the sinonasal tract. *Archives of Pathology & Laboratory Medicine*.

[11] La T. H., Meyers P. A., Wexler L. H. (2006). Radiation therapy for Ewing's sarcoma: results from Memorial Sloan-Kettering in the modern era. *International Journal of Radiation Oncology∗Biology∗Physics*.

[12] Womer R. B., West D. C., Krailo M. D. (2012). Randomized controlled trial of interval-compressed chemotherapy for the treatment of localized ewing sarcoma: a report from the children's oncology group. *Journal of Clinical Oncology*.

